# Supplementary material for: The Impact of the Oil Phase Selection on Physicochemical Properties, Long-Term Stability, In Vitro Performance and Injectability of Curcumin-Loaded PEGylated Nanoemulsions
Source: Pharmaceutics. 2022 Aug 10;14(8):1666. doi: 10.3390/pharmaceutics14081666 (PMC9415641; doi:10.3390/pharmaceutics14081666)
Supplement: Supplementary file 1 [file pharmaceutics-14-01666-s001.zip › pharmaceutics-1815554-supplementary.pdf]

Supplementary material

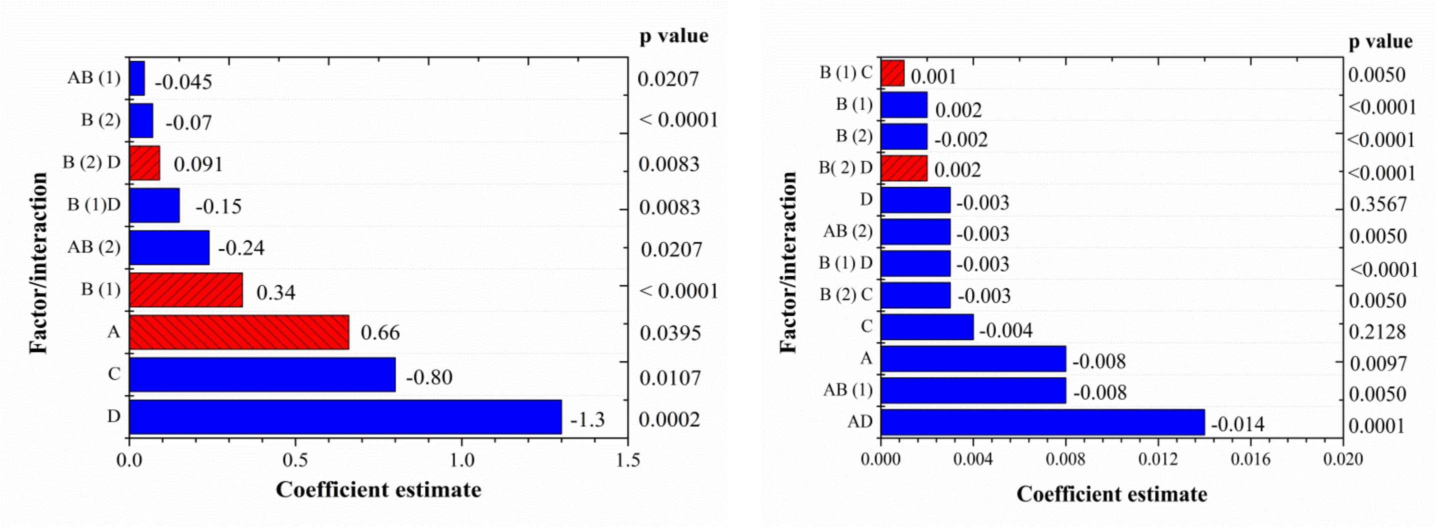

(a) (b)

Figure S1. Plots for: a) Z-ave – mean droplet size and b) PDI - polydispersity index, with the coefficient and p values for the factors and their interactions. A: PEG-PL type; B: PEG-PL concentration; C- oil type and D: the presence of curcumin.

Table S1. Results of laser diffraction (LD) measurements for soybean oil formulations stored at room temperature for two years.

Formulations	d (10) (nm)	d (50) (nm)	d (90) (nm)	D [4,3] (nm)
**PS**	78	116	173	122
**CS**	78	117	173	122
**S21**	76	114	173	120
**CS21**	76	113	168	118
**S23**	74	109	163	115
**CS23**	75	112	166	117
**S26**	74	109	163	115
**CS26**	75	111	165	117
**S51**	76	113	169	118
**CS51**	76	113	168	118
**S53**	74	109	164	115
**CS53**	75	112	167	117
**S56**	73	106	159	112
**CS56**	76	112	166	117

Table S2. Results of laser diffraction (LD) measurements for fish oil formulations stored at room temperature for two years.

Formulations	d (10) (nm)	d (50) (nm)	d (90) (nm)	D [4,3] (nm)
**PF**	224	309	421	317
**CF**	78	116	173	121
**F21**	74	109	163	115
**CF21**	76	112	167	118
**F23**	73	106	159	112
**CF23**	76	114	171	120
**F26**	74	109	162	114
**CF26**	76	112	167	117
**F51**	76	112	167	118
**CF51**	76	113	168	118
**F53**	79	122	207	1378
**CF53**	76	112	167	117
**F56**	79	120	195	1897
**CF56**	76	112	167	117

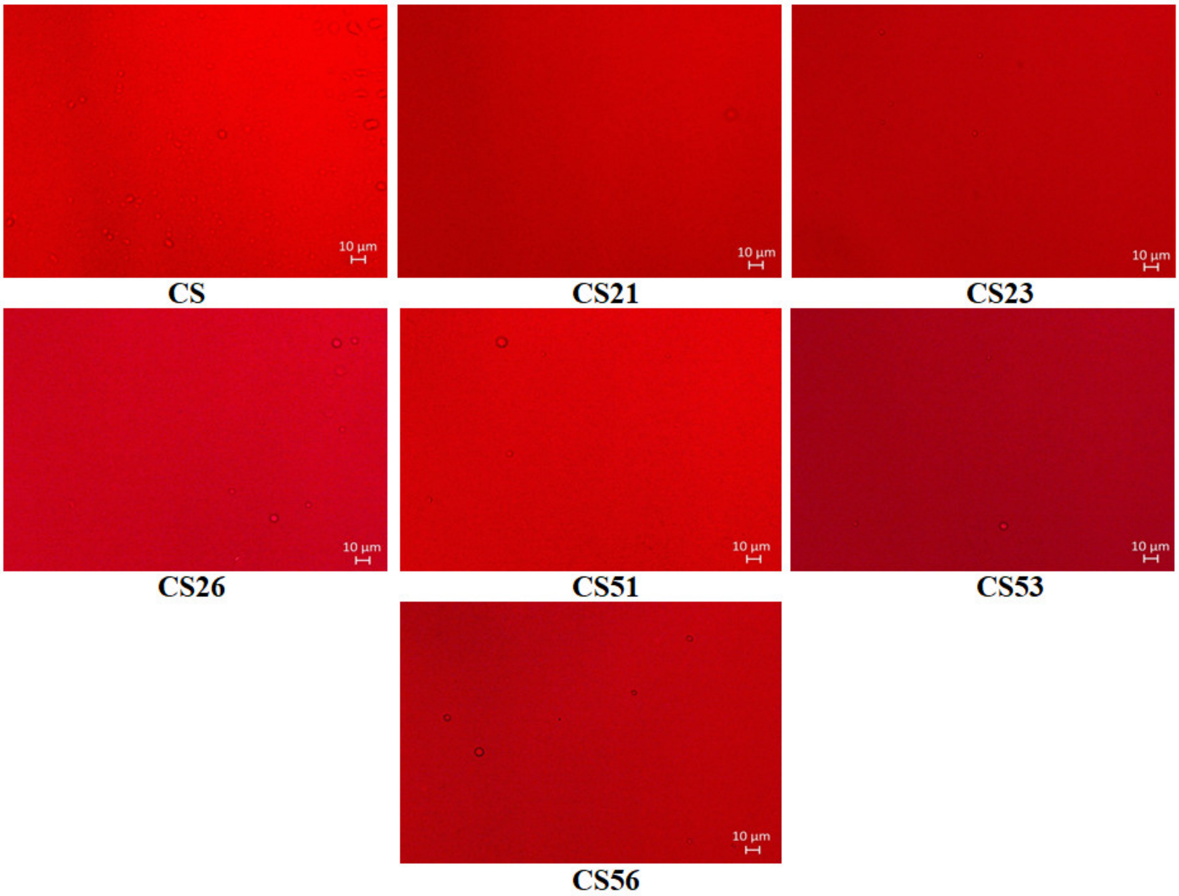

Figure S2. Polarization microscopy images of the curcumin-loaded soybean oil NEs taken after two years of storage.

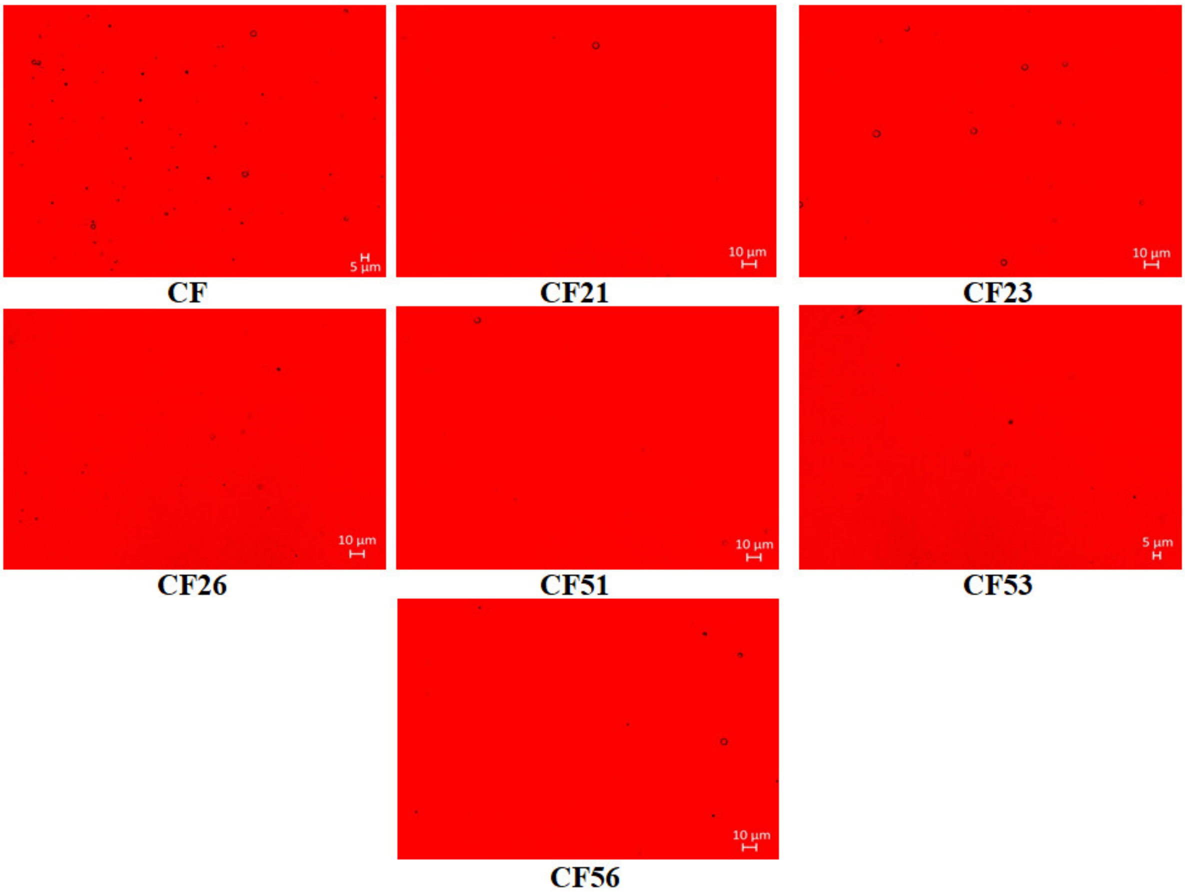

Figure S3. Polarization microscopy images of the curcumin-loaded fish oil NEs taken after two years of storage.

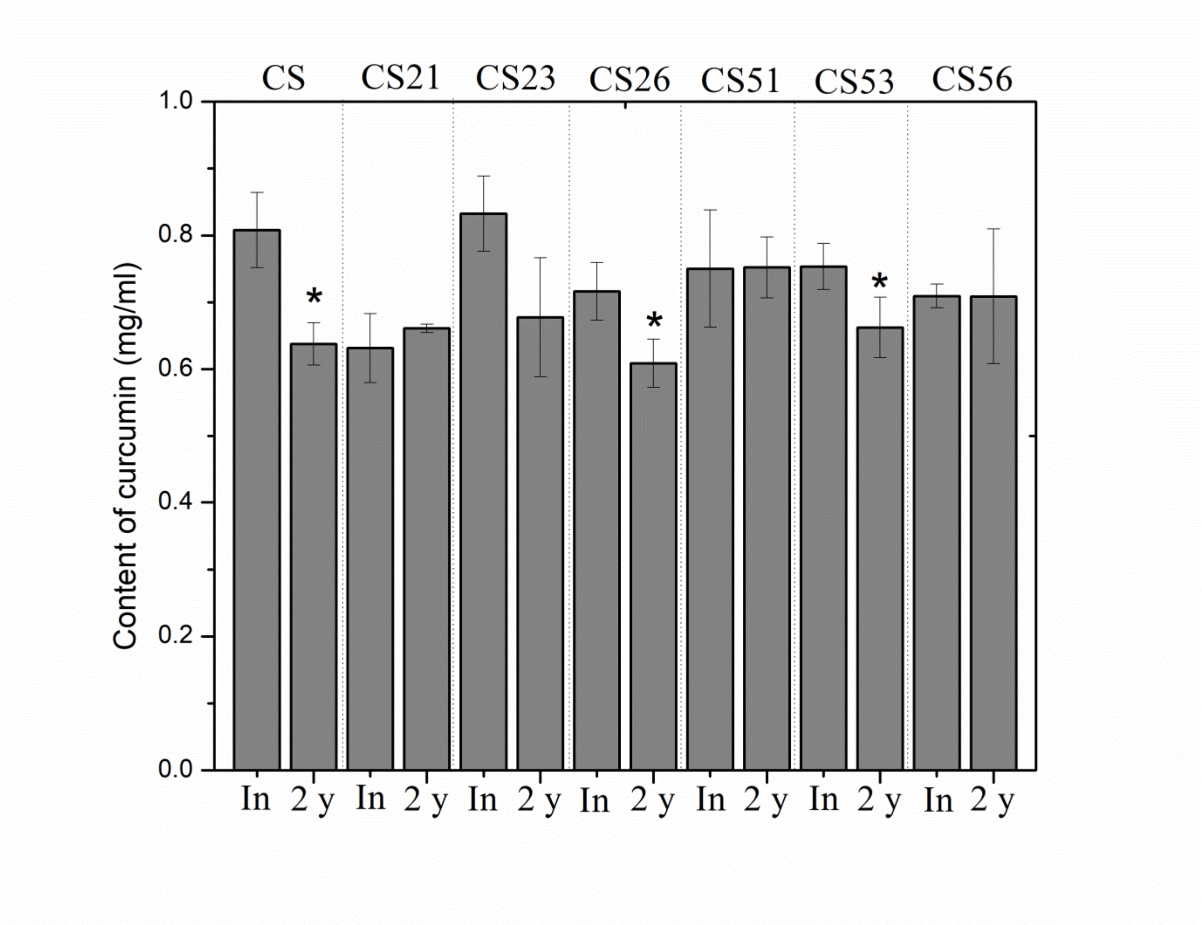

Figure S4. Content of curcumin in soybean oil nanoemulsions measured initially (In) and after two years of storage (2y); Values are shown as means ± sd (n = 3); *, $p < 0.05$, compared to the initially measured values.

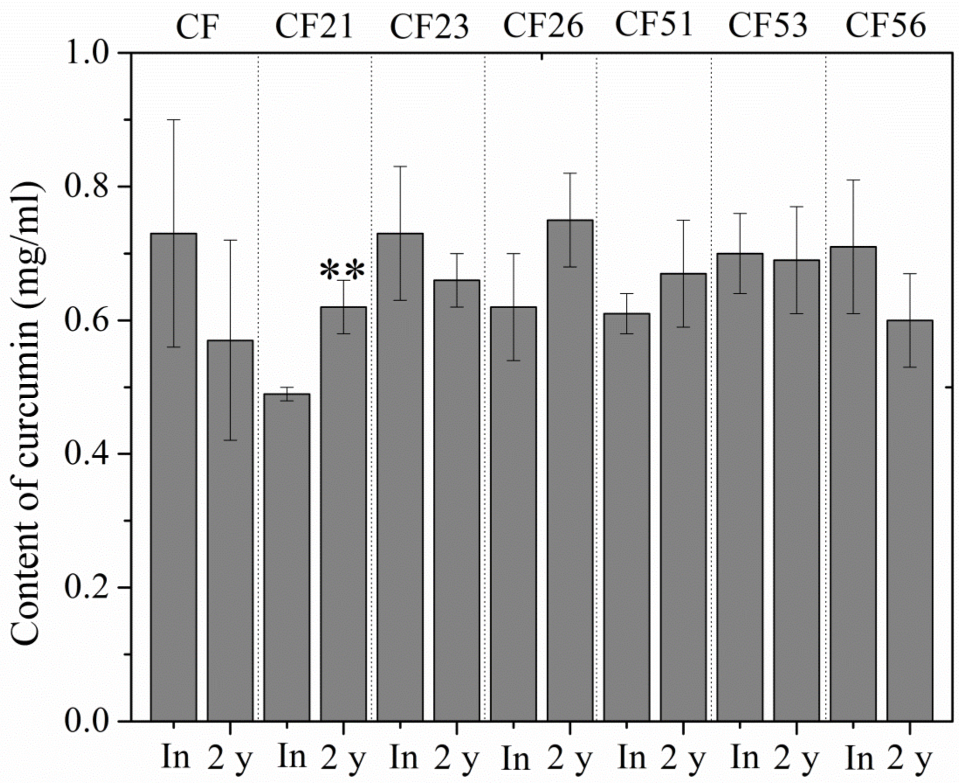

Figure S5. Content of curcumin in fish oil nanoemulsions measured initially (In) and after two years of storage (2y); Values are shown as means ± sd (n = 3); **, $p < 0.01$, compared to the initially measured values.

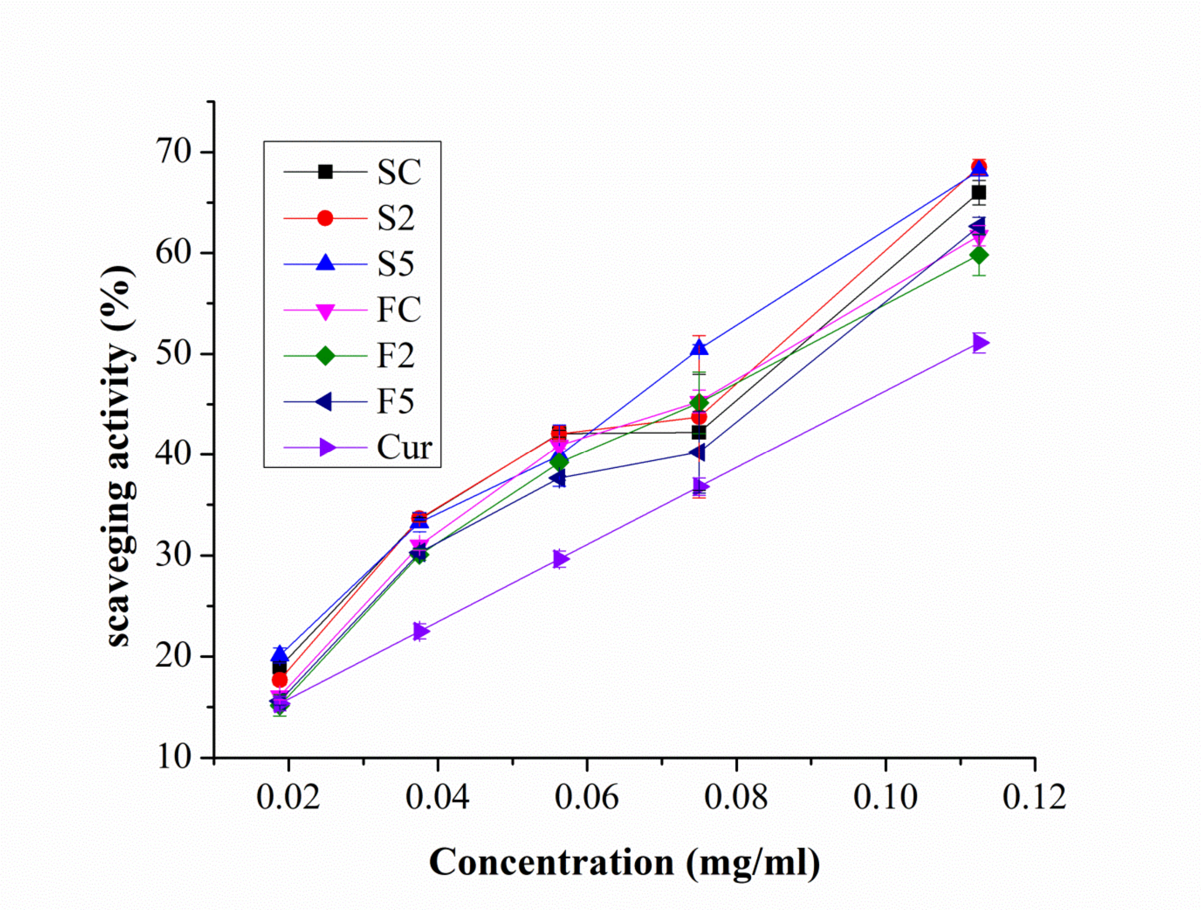

Figure S6. DPPH assay results for freshly prepared nanoemulsions with soybean oil (SC, S21, S51), fish oil (FC, F21, F51) and pure curcumin (Cur).

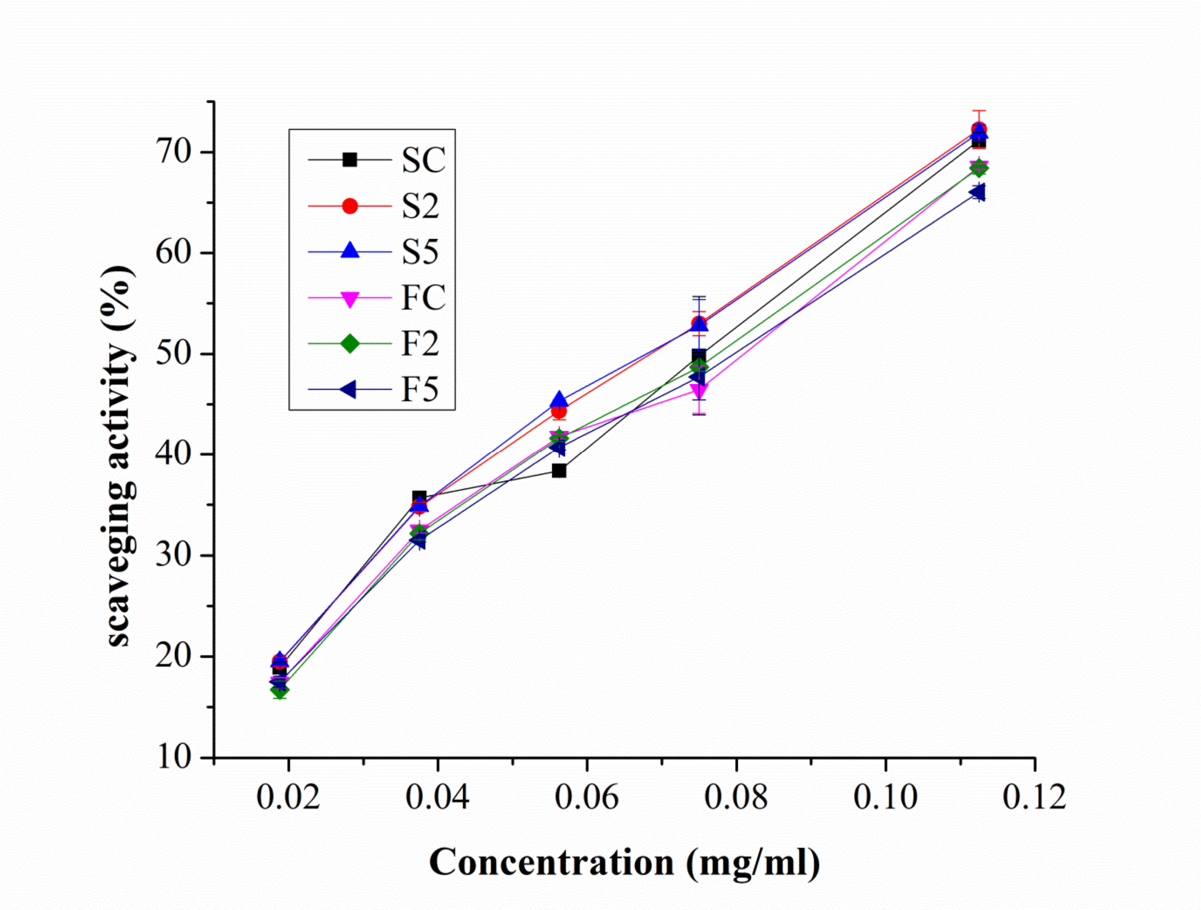

Figure S7. DPPH assay results for nanoemulsions with soybean oil (SC, S21, S51) and fish oil (FC, F21, F51) after two years of storage.

Table S3. Dissolution modelling for non-PEGylated formulations.

	Formulations	
	CS	CF
Zero order	**K = 0.100**	K = 0.250
	**R^2 = 0.9918**	R^2 = 0.9930
	**R^2_{adj} = 0.9918**	R^2_{adj} = 0.9930
	**AIC = 8.7911**	AIC = 17.8459
First order	K = 0.001	K = 0.003
	R^2 = 0.9907	R^2 = 0.9581
	R^2_{adj} = 0.9907	R^2_{adj} = 0.9851
	AIC =10.0073	AIC = 23.3072
Higuchi	K = 1.198	K = 3.031
	R^2 = 0.8009	R^2 = 0.8278
	R^2_{adj} = 0.8009	R^2_{adj} = 0.8278
	AIC =28.3909	AIC = 38.0503
Baker-Lonsdale	K = 0.000	K = 0.000
	R^2 = 0.7883	R^2 = 0.7914
	R^2_{adj} = 0.7883	R^2_{adj} = 0.7914
	AIC =28.7596	AIC = 39.2015
Korsmeyer-Peppas	K = 0.095	**K = 0.325**
	N = 1.013	**N = 0.952**
	R^2 = 0.9925	**R^2 = 0.9945**
	R^2_{adj} = 0.9906	**R^2_{adj} = 0.9931**
	AIC = 10.2221	**AIC = 18.5235**
Hixson-Crowell	K = 0.000	K = 0.001
	R^2 = 0.9916	R^2 = 0.9927
	R^2_{adj} = 0.9916	R^2_{adj} = 0.9927
	AIC = 9.2999	AIC = 19.0848

Table S4. Dissolution modelling for PEGylated formulations containing PEG2000-DSPE.

	Formulations					
	CS21	CS23	CS26	CF21	CF23	CF26
Zero order	K = 0.101	**K = 0.105**	K = 0.082	K = 0.282	**K = 0.382**	**K = 0.324**
	R^2 = 0.9894	**R^2 = 0.9907**	R^2 = 0.9640	R^2 = 0.9911	**R^2 = 0.9876**	**R^2 = 0.9956**
	R^2_{adj} = 0.9894	**R^2_{adj} = 0.9907**	R^2_{adj} = 0.9640	R^2_{adj} = 0.9911	**R^2_{adj} = 0.9876**	**R^2_{adj} = 0.9956**
	AIC = 10.4473	**AIC =10.5040**	AIC = 14.5805	AIC = 20.6449	**AIC = 26.3692**	**AIC = 18.5561**
First order	K = 0.001	K = 0.001	K = 0.001	K = 0.004	K = 0.006	K = 0.005
	R^2 = 0.9860	R^2 = 0.9896	R^2 = 0.9546	R^2 = 0.9471	R^2 = 0.9434	R^2 = 0.9535
	R^2_{adj} = 0.9860	R^2_{adj} = 0.9896	R^2_{adj} = 0.9546	R^2_{adj} = 0.9471	R^2_{adj} = 0.9434	R^2_{adj} = 0.9535
	AIC = 12.7480	AIC = 10.9469	AIC = 16.4633	AIC = 31.9855	AIC = 36.5120	AIC = 33.4300
Higuchi	K = 1.205	K = 1.254	K = 0.960	K = 3.351	K = 4.627	K = 3.904
	R^2 = 0.7860	R^2 = 0.7996	R^2 = 0.7129	R^2 = 0.7756	R^2 = 0.8206	R^2 = 0.8086
	R^2_{adj} = 0.7860	R^2_{adj} = 0.7996	R^2_{adj} = 0.7129	R^2_{adj} = 0.7756	R^2_{adj} = 0.8206	R^2_{adj} = 0.8086
	AIC = 29.1103	AIC = 28.9471	AIC = 28.8838	AIC = 41.5504	AIC = 43.5013	AIC = 41.9425
Baker-Lonsdale	K = 0.000	K = 0.000	K = 0.000	K = 0.000	K = 0.000	K = 0.000
	R^2 = 0.7732	0.7863R^2 =	R^2 = 0.7023	R^2 = 0.7317	R^2 = 0.7565	R^2 = 0.7560
	R^2_{adj} = 0.7732	R^2_{adj} =0.7863	R^2_{adj} = 0.7023	R^2_{adj} = 0.7317	R^2_{adj} = 0.7565	R^2_{adj} = 0.7560
	AIC = 29.4613	AIC = 29.3340	AIC = 29.1155	AIC = 42.6221	AIC = 45.3473	AIC = 43.4036
Korsmeyer-Peppas	**K = 0.080**	K = 0.100	**K = 0.031**	**K = 0.178**	K = 0.494	K = 0.315
	**N = 1.050**	N = 1.014	**N = 1.300**	**N = 1.096**	N = 0.959	N = 1.008
	**R^2 = 0.9913**	R^2 = 0.9917	**R^2 = 0.9924**	**R^2 = 0.9965**	R^2 = 0.9901	R^2 = 0.9958
	**R^2_{adj} = 0.9891**	R^2_{adj} = 0.9896	**R^2_{adj} = 0.9905**	**R^2_{adj} = 0.9956**	R^2_{adj} = 0.9876	R^2_{adj} = 0.9948
	**AIC = 9.7759**	AIC = 11.8500	**AIC = 7.2300**	**AIC = 18.4773**	AIC = 27.5574	AIC = 20.2089
Hixson-Crowell	K = 0.000	K = 0.000	K = 0.000	K = 0.001	K = 0.002	K = 0.001
	R^2 = 0.9877	R^2 = 0.9906	R^2 = 0.9580	R^2 = 0.9660	R^2 = 0.9695	R^2 = 0.9746
	R^2_{adj} = 0.9877	R^2_{adj} = 0.9906	R^2_{adj} = 0.9580	R^2_{adj} = 0.9660	R^2_{adj} = 0.9695	R^2_{adj} = 0.9746
	AIC = 11.8910	AIC = 10.4372	AIC = 15.8103	AIC = 29.0151	AIC = 32.7449	AIC = 29.7493

Table S5. Dissolution modelling for PEGylated formulations containing PEG5000-DPPE.

	Formulations					
	CS51	CS53	CS56	CF51	CF53	CF56
Zero order	K = 0.120<br>R^2 = 0.9614<br>R^2_{adj} = 0.9614<br>AIC = 21.0680	K = 0.414<br>R^2 = 0.9752<br>R^2_{adj} = 0.9752<br>AIC =20.6748	K = 0.147<br>R^2 = 0.9767<br>R^2_{adj} = 0.9767<br>AIC = 20.7972	**K = 0.308**<br>**R^2 = 0.9929**<br>**R^2_{adj} = 0.9929**<br>**AIC = 21.5793**	**K = 0.374**<br>**R^2 = 0.9848**<br>**R^2_{adj} = 0.9848**<br>**AIC = 28.5973**	**K = 0.434**<br>**R^2 = 0.9931**<br>**R^2_{adj} = 0.9931**<br>**AIC =25.2423**
First order	K = 0.001<br>R^2 = 0.9435<br>R^2_{adj} = 0.9435<br>AIC = 23.5581	K = 0.002<br>R^2 = 0.9559<br>R^2_{adj} = 24.2330<br>AIC = 24.2330	K = 0.002<br>R^2 = 0.9581<br>R^2_{adj} = 0.9581<br>AIC = 24.3848	K = 0.004<br>R^2 = 0.9686<br>R^2_{adj} = 0.9686<br>AIC = 30.1040	K = 0.006<br>R^2 = 0.9176<br>R^2_{adj} = 0.9176<br>AIC = 38.9857	K = 0.007<br>R^2 = 0.8965<br>R^2_{adj} = 0.8965<br>AIC = 41.9234
Higuchi	K = 1.389<br>R^2 = 0.6986<br>R^2_{adj} = 0.6986<br>AIC = 34.0295	K = 1.648<br>R^2 = 0.7237<br>R^2_{adj} = 0.7237<br>AIC = 35.3250	K = 1.713<br>R^2 = 0.7298<br>R^2_{adj} = 0.7298<br>AIC = 35.5760	K = 3.728<br>R^2 = 0.8196<br>R^2_{adj} = 0.8196<br>AIC = 40.9533	K = 4.457<br>R^2 = 0.7780<br>R^2_{adj} = 0.7780<br>AIC = 44.9222	K = 5.202<br>R^2 = 0.7951<br>R^2_{adj} = 0.7951<br>AIC = 46.0397
Baker-Lonsdale	K = 0.000<br>R^2 = 0.6826<br>R^2_{adj} = 0.6826<br>AIC = 34.3458	K = 0.000<br>R^2 = 0.7045<br>R^2_{adj} = 0.7045<br>AIC = 35.7292	K = 0.000<br>R^2 = 0.7099<br>R^2_{adj} = 0.7099<br>AIC = 36.0033	K = 0.000<br>R^2 = 0.7716<br>R^2_{adj} = 0.7716<br>AIC = 42.3767	K = 0.000<br>R^2 = 0.7147<br>R^2_{adj} = 0.7147<br>AIC = 46.4540	K = 0.001<br>R^2 = 0.7151<br>R^2_{adj} = 0.7151<br>AIC = 48.0304
Korsmeyer-Peppas	**K = 0.031**<br>**N = 1.307**<br>**R^2 = 0.9899**<br>**R^2_{adj} = 0.9874**<br>**AIC = 15.5387**	**K = 0.044**<br>**N = 1.227**<br>**R^2 = 0.9920**<br>**R^2_{adj} = 0.9900**<br>**AIC = 15.8758**	**K = 0.051**<br>**N = 1.201**<br>**R^2 = 0.9903**<br>**R^2_{adj} = 0.9879**<br>**AIC = 17.2340**	K = 0.366<br>N = 0.971<br>R^2 = 0.9942<br>R^2_{adj} = 0.9927<br>AIC = 22.1855	K = 0.294<br>N = 1.061<br>R^2 = 0.9877<br>R^2_{adj} = 0.9846<br>AIC = 29.4679	K = 0.377<br>N = 1.034<br>R^2 = 0.9941<br>R^2_{adj} = 0.9927<br>AIC = 25.9541
Hixson-Crowell	K = 0.000<br>R^2 = 0.9501<br>R^2_{adj} = 0.9501<br>AIC = 22.7485	K = 0.001<br>R^2 = 0.9632<br>R^2_{adj} = 0.9632<br>AIC = 23.1150	K = 0.001<br>R^2 = 0.9653<br>R^2_{adj} = 0.9653<br>AIC = 23.2384	K = 0.001<br>R^2 = 0.9842<br>R^2_{adj} = 0.9842<br>AIC = 25.5464	K = 0.002<br>R^2 = 0.9483<br>R^2_{adj} = 0.9483<br>AIC = 36.1667	K = 0.002<br>R^2 = 0.9359<br>R^2_{adj} = 0.9359<br>AIC = 39.0479

Table S6. Maximum injection force (N) for soybean and fish oil nanoemulsions.

Formulations	Maximum injection force (Fmax)
CS	19.65 ± 1.54
CS21	22.94 ± 0.26
CS23	27.66 ± 1.00
CS26	29.34 ± 1.20
CS51	25.21 ± 0.33
CS53	28.58 ± 2.34
CS56	34.60 ± 2.58
CF	19.58 ± 1.55
CF21	23.39 ± 2.45
CF23	26.53 ± 2.51
CF26	30.57 ± 0.45
CF51	24.35 ± 0.46
CF53	29.06 ± 2.37
CF56	35.81 ± 3.27

Values are shown as means ± sd (n = 3).
